# The effectiveness of knowledge translation strategies used in public health: a systematic review

**DOI:** 10.1186/1471-2458-12-751

**Published:** 2012-09-07

**Authors:** Rebecca LaRocca, Jennifer Yost, Maureen Dobbins, Donna Ciliska, Michelle Butt

**Affiliations:** 1 McMaster University Medical Centre, 1200 Main St. West, Hamilton, Ontario, Canada; 2 School of Nursing, McMaster University, 1280 Main St. West, Hamilton, Ontario, Canada

**Keywords:** Knowledge translation, Public health, Research utilisation, Research transfer

## Abstract

**Background:**

Literature related to the effectiveness of knowledge translation (KT) strategies used in public health is lacking. The capacity to seek, analyze, and synthesize evidence-based information in public health is linked to greater success in making policy choices that have the best potential to yield positive outcomes for populations. The purpose of this systematic review is to identify the effectiveness of KT strategies used to promote evidence-informed decision making (EIDM) among public health decision makers.

**Methods:**

A search strategy was developed to identify primary studies published between 2000–2010. Studies were obtained from multiple electronic databases (CINAHL, Medline, EMBASE, and the Cochrane Database of Systematic Reviews). Searches were supplemented by hand searching and checking the reference lists of included articles. Two independent review authors screened studies for relevance, assessed methodological quality of relevant studies, and extracted data from studies using standardized tools.

**Results:**

After removal of duplicates, the search identified 64, 391 titles related to KT strategies. Following title and abstract review, 346 publications were deemed potentially relevant, of which 5 met all relevance criteria on full text screen. The included publications were of moderate quality and consisted of five primary studies (four randomized controlled trials and one interrupted time series analysis). Results were synthesized narratively. Simple or single KT strategies were shown in some circumstances to be as effective as complex, multifaceted ones when changing practice including tailored and targeted messaging. Multifaceted KT strategies led to changes in knowledge but not practice. Knowledge translation strategies shown to be less effective were passive and included access to registries of pre-processed research evidence or print materials. While knowledge brokering did not have a significant effect generally, results suggested that it did have a positive effect on those organizations that at baseline perceived their organization to place little value on evidence-informed decision making.

**Conclusions:**

No singular KT strategy was shown to be effective in all contexts. Conclusions about interventions cannot be taken on their own without considering the characteristics of the knowledge that was being transferred, providers, participants and organizations.

## Background

Globally, and at all levels of health care, health systems fail to use research evidence optimally [1]. This gap results in negative effects, such as a reduction in both quantity and quality of life [2] and inefficient use of limited health care resources [1,3]. As political and societal pressures to use research evidence in decision making continue to rise, there is increased interest in the concept of knowledge translation [4]. Knowledge translation (KT) is defined by the Canadian Institutes of Health Research as "*a dynamic and iterative process that includes the synthesis, dissemination, exchange and ethically sound application of knowledge to improve the health of populations, provide more effective health services and products and strengthen the health care system"*[4]. KT strategies are used in public health to promote evidence-informed decision making (EIDM). EIDM refers to incorporating the best available research evidence into public health policy and program decision making [5]. The rationale for engaging in EIDM is the belief that optimal patient and population health outcomes will result [6].

Translating best available research evidence into programmatic change is a complex process [7]. Multiple barriers to EIDM exist at different levels. Examples of these include but are not limited to: the health care system itself (lack of financial incentives); health care organizations (limited access to research evidence, lack of equipment); health care teams (existing standards may not be in line with recommended practice); individual health care professionals (lack of knowledge, attitudes and skills in critically appraising and using evidence from the literature, lack of time and resistance to change); and patients (poor compliance to recommendations) [1,5].

Despite KT strategies to overcome such barriers in public health, literature related to how to effectively promote and facilitate these strategies are lacking [8]. To our knowledge, only one related systematic review in public health was completed by Stone and colleagues [9]. Inclusive of primary studies published prior to 2000, Stone and colleagues evaluated KT intervention components classified as provider reminders, provider feedback, provider education, provider financial incentive, and organizational change to increase screening related to immunisation and cancer. The KT strategy found to be most effective for improved use of adult preventative services by providers was organizational change. The KT strategy found to be least effective was provider feedback [9].

While important evidence can come from a variety of sources, evidence from best available research findings should be one of the essential components of the policy development and decision making processes that occur within public health agencies. There is however a paucity of literature regarding how to facilitate this process specifically in the public health setting [5,7,8,10]. The objective of this review is to address which KT strategies are most effective among practitioners, managers and policy makers to promote the use of research evidence in public health settings.

## Methods

### Data sources and search strategy for identification of studies

Multiple and competing terms exist to describe the study of implementing research findings into practice [11]; it is referred to as “KT” in this review. Terms for related concepts are often used interchangeably, and definitions are unclear [3,12] which makes information retrieval related to the field of KT very difficult [11]. As such, the search strategy for this systematic review was developed by Dr. Ann McKibbon, of the Health Information Research Unit, Department of Clinical Epidemiology and Biostatistics at McMaster University. In addition to using the two key concept categories of 'public health', and knowledge translation'; related KT terms identified in the cross-sectional study by Dr. Ann McKibbon and colleagues [11] with high and medium discriminatory power were used to identify potentially relevant studies.

The search strategy, which aimed to find both published and unpublished studies, was limited to the English language and restricted to the dates 2000 to 2010 inclusive (see Additional File 1). These dates were chosen for three different reasons. The first and primary reason was that due to the challenges associated with retrieving articles related to the field of KT described above, the assumption was made that our purposely broad and inclusive literature search of ten years would produce an extremely large yield. Reviewing an extremely large yield of references in a systematic and unbiased manner is challenging and can be extensive in terms of resources including time. The assumption of a large yield resulting from our search was also based on the review authors' prior experiences locating related KT literature.

The second reason was based on the likelihood of very few public health relevant studies existing prior to the year 2000. While evidence exists to support the need for KT, very little evidence exists that measures the impact of KT interventions [13]. Only one related public health study, a systematic review by Stone and colleagues [9] was found in the search for the current review. This systematic review included primary studies published prior to 2000 and their primary limitation reported was the quantity of existing original studies. In addition, unlike the interest and resources long committed to evidence-based medicine, EIDM only became part of the public health sector's lexicon in the 1990's [14]. It is therefore reasonable to assume that the literature prior to 2000 would primarily have a focus on medical or acute care professionals , which are not the targeted group in this review. Similarly, while the term KT has been used in adult education research in the 1950s, only recently has KT become used in the context of implementing best research evidence and was more recognized and studied as such in the late 1990s and early 2000s [11].

The third and final reason the search strategy was limited to studies since 2000 was to obtain related literature that would reflect the public health system today since context weighs heavily on the effectiveness of different KT interventions [14]. The context-specific nature of KT adds an additional degree of complexity in evaluating the impact of KT strategies [15]. The review authors therefore felt it was important to locate research evidence that may be more generalizable to the environments public health practitioners are working in today.

The Effective Practice and Organisation of Care Group (EPOC) is a group within the Cochrane Collaboration (available at: http://epoc.cochrane.org/). After searching their database and finding very few studies related to KT and public health, the following databases were searched on April 12, 2010: CINAHL, Medline, EMBASE, and the Cochrane Database of Systematic Reviews from 2000 to 2010. Methodological study filters from EPOC were utilized in order to search for relevant study designs (see inclusion criteria). The electronic searches were supplemented by checking the reference lists of included articles and hand searching online databases of research relevant to KT or public health including *Knowledge Translation + (available at:*http://plus.mcmaster.ca/kt/) and *Public Health + (available at:*http://www.nccmt.ca/public_health_plus/all/1/list-eng.html). Searches were also supplemented by checking conference proceedings and grey literature including: Canadian Public Health Association, Research Transfer Network of Alberta, Knowledge Exchange in Public Health, National Institutes of Health, and the 2010 Public Health Policy Conference. Contact was also made with study authors to identify additional studies.

### Study selection

#### Type of participants

Studies directed towards health practitioners in a public health or community setting were included in this review. The recipients of the strategy or intervention was all practitioners, including allied health professionals, involved in public health and health promotion services. Public health services exist to help persons achieve better health and well-being by promoting good health, preventing chronic disease and injury, and protecting persons from infectious disease and other threats to their health [16]. Its focus is therefore on prevention, opposed to treating existing illness. Examples of public health services include vaccinations and immunizations, health promotion of preventable illness such as childhood obesity and diabetes, pandemic preparedness, and injury prevention. Inclusion criteria included studies directed towards health professionals involved in the delivery of these or additional preventative services. This included but was not limited to nurses, physicians, social workers, occupational therapists, dietitians, administrators, policy and decision makers whose focus was on preventative care.

If the practitioners in the studies were decided by the review authors to be involved in activities focused on clinical care, for example the treatment of an existing illness such as cancer, opposed to activities focused on prevention and health promotion described above; they were not considered public health practitioners and excluded from this review. Studies where participants were students learning in a school setting and practitioners in the primary care, tertiary or community health settings focused on clinical care were excluded.

#### Type of intervention

Any KT strategy directed towards the providers and aimed at building capacity for EIDM by promoting or facilitating the utilization of research evidence were included in this review. Evidence informed capacity can be defined as the ability of practitioners to draw on, filter and amplify appropriate research evidence [17]. Examples of eligible KT interventions included, but were not limited to, the use of education, reminders, audit and feedback, knowledge brokers, tailored messaging or champions.

#### Type of outcomes

This review focused on a variety of possible outcomes that can be categorized to include change in knowledge, skill or practice. A change in knowledge included any concrete change in knowledge or understanding. For example, a positive change in knowledge or understanding could be observed through an improved test score evaluating respondents' knowledge of EIDM concepts taught in the KT intervention. A change in attitude was not included because the review authors felt that a change in attitude was not a concrete measure of change in knowledge.

A change in skill may include skills in commissioning and interpreting evidence. For example, an increased ability to locate best available evidence within appropriate databases. A change in practice was defined as the concrete application of knowledge. This could be an actual change in behaviour or practice including a change in decision making used to influence program planning or influence policy. Changes in practice could be observed through research evidence being referenced or utilized in public health policy, practice, program or guideline development, or changes in public health policy and practice [5].

#### Type of studies

Given many KT interventions are tested in real-life, practice based settings it is not always feasible to evaluate them using randomized controlled trials (RCT)s [18]. Therefore study designs accepted for EPOC systematic reviews were included in this review: practitioner randomized controlled trials, cluster randomized controlled trials, non-randomized cluster controlled trials, controlled before and after studies, and interrupted time series designs. Relevant systematic reviews were also included. Qualitative and mixed method study designs were excluded.

### Screening, quality assessment and data extraction

#### Screening

The web based application DistillerSR (available at: http://systematic-review.net/) was used to manage all references and assist in the review process. After duplicate articles were removed, the titles and abstracts from all search strategies were imported into DistillerSR and screened independently by the primary researcher (RL) and one of four other reviewers. Studies deemed to be potentially relevant by either reviewer were retrieved for full-text review. The full-text was assessed for relevance independently by two review authors: the primary author (RL) and the corresponding author (JY). Agreement needed to be reached by both authors for inclusion.

#### Quality assessment

Studies deemed to be relevant were then assessed for methodological quality by two independent review authors (RL, JY) using standardized tools. All studies that met the inclusion criteria were included in the review, regardless of methodological quality (See Additional file 2). For RCTs, the review authors (RL, JY) conducted a domain-based evaluation of the risk of bias within each included study using a tool recommended by the Cochrane Collaboration [19]. The following criteria were addressed independently by two blinded review authors: sequence generation, allocation concealment, blinding, incomplete outcome data, selective outcome reporting and ‘other issues.’ Sequence generation, allocation concealment and selective outcome reporting were addressed by a single entry for each study while blinding and incomplete outcome data assessments were made separately for different outcomes [19]. For designs that utilized an interrupted time series design, risk of bias was assessed using EPOC's Risk of Bias tool (available at: http://www.epoc.cochrane.org).

#### Data extraction

Data were extracted using a tool developed by the Effective Public Health Practice Project [20]. The data extraction tool has been pilot tested and refined over use in more than 20 reviews. The primary review author conducted the data extraction and it was reviewed by a second review author (JY). Specific details including characteristics of included studies, details about the intervention, population, follow-up period, attrition rates, study methods and outcomes significant to the review were also extracted. Outcome data prior to the intervention and at the last follow up date were extracted. Authors were contacted for missing data or when clarification was required. Disagreements that occurred during the screening, quality assessment, or data extraction were discussed until consensus was achieved. A third review author was consulted if consensus could not be reached

## Results

**Figure 1:**
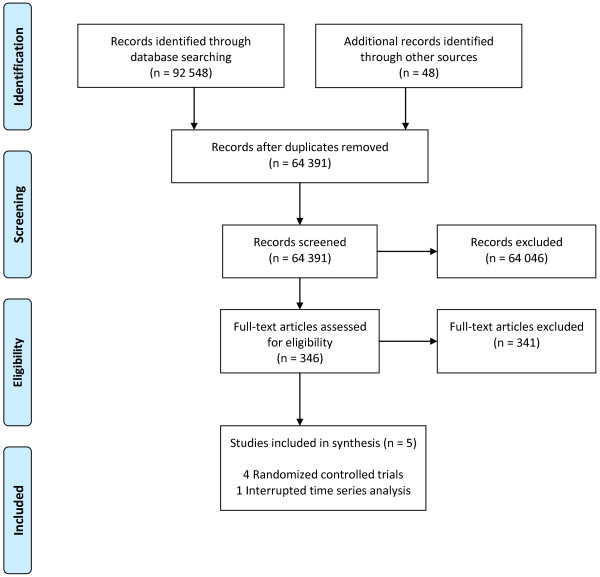
**Flow Diagram.** Flow diagram of systematic review to identify eligible studies.

The search strategy identified 92,548 titles related to KT interventions; 64,391 after duplicate articles were removed (Figure 1).

Of the 64, 391 articles, 346 articles were deemed potentially relevant following title and abstract review. Titles were most often considered not relevant because the KT strategy was not implemented in a public health or community setting or because the KT strategy was not directed towards public health practitioners. Of the 346 articles retrieved for full text review, 5 primary studies met all relevance criteria and were included in this review. The most common reasons full-text studies were judged as not relevant were that the intervention was not a knowledge translation strategy or information on relevant outcomes were not reported. Of the 5 studies, four were RCTs [5,21-23], and one was a time series analysis [24]. Additional File 3 provides the characteristics of included studies. The systematic review by Stone and colleagues [9] was not included because the individual studies included in their review were all published between 1979 to 1999 which did not meet our inclusion criteria.

### Participants and settings

Of the five included primary studies one was conducted in the United States [22], two were conducted in Canada [5,21], and the remaining two conducted in European countries, Norway [23], and England [24]. The unit of allocation was done by individual [23] and by organization or site [5,21,22,24]. The smallest sample size was 34 public health practitioners [21] and the largest sample size included 188 practitioners [22]. All of the studies evaluated the outcome, change in practice, three studies evaluated changes in knowledge [21-23] and none evaluated changes in skill level. Four of the five studies evaluated outcomes immediately following the intervention, with one of the studies evaluating outcomes six months post intervention [22].

KT strategies were aimed at a variety of public health professionals involved in public or community prevention orientated coalitions from a range of public health disciplines including mental health [21,24], preventative adolescent substance abuse services [22], healthy body weight promotion [5] and immunization and cancer screening prevention [23]. Strategies were targeted at community providers employed by public health departments, community agencies and policy making bodies including school personnel, social workers, registered nurses, physicians, program managers, coordinators or directors.

### Knowledge translation strategies

Although the universal objective of studies was to build capacity for practitioners involved in using evidence, KT strategies varied considerably with none of the primary studies evaluating the same KT strategy. KT strategies evaluated in the five studies included: educational sessions involving peer development [21,24] and workshops [23]; dissemination channels including print, CD-ROM, and Internet [22]; technical assistance and staff training from consultants with varying levels of interaction and supervision [5,23]; and web-based services such as databases, information services and discussion lists [23], and registries of pre-processed research evidence or online tailored and targeted messaging [5].

### Effect of the intervention

Due to the variability in the type of KT strategies and implementation of these strategies, as well as differences in data collection between the included studies it is difficult to estimate the magnitude of the impact. With such variation, a meta-analysis was not warranted. Therefore the following provides a narrative synthesis of the results which should be interpreted cautiously.

#### Change in knowledge

Three of the five studies evaluated changes in knowledge all of which were RCTs [21-23]. Two of the three studies [22,23] found statistically significant between group differences (Table 1). Di Noia et al. [22] disseminated adolescent substance abuse prevention program materials to school personnel, community providers and policy makers through pamphlet, CD-ROM, and Internet channels. At 6 month follow-up, respondents who received prevention materials disseminated via CD-ROM and Internet showed significantly greater knowledge of where to locate drug abuse prevention findings and materials compared to those who received printed pamphlets (p < 0.05) [22]. Bonferroni post hoc comparisons revealed differences in favour of respondents using the Internet (p < 0.05) [22]. Forsetlund et al. [23] tested a multi-faceted strategy designed to lead participants through steps outlined in Rogers' model of innovation diffusion. The strategy for the intervention group included an 11 course skill building workshop on evidence-based public health involving small group problem-based activities and discussion, goal setting, access to web-based information services (inclusive of a question and answer service, discussion list, and ongoing support services), and 3 newsletters. The control group received access to library services only. Statistically significant differences were found between the two groups for both concept (p = 0.001) and source knowledge scores (p < 0.01). Sensitivity analysis was conducted and a significant difference remained even after assigning the control group's mean value (1.1) to missing values in both groups. When assigning the control group's lowest value (0) to replace missing data in both groups, the results for concept knowledge became non-significant [23].

**Table 1:** Outcomes table for change in knowledge

**Randomized controlled trials (3)**							
**Study**	**Measurement period**	**Study population**	**groups**	**Baseline**	**Follow up**	**Overall effect**	**Measurement**
Barwick 2009	Baseline	34 Child & youth mental health practitioners		Mean Score:	Mean Score:	F= 2.37	CAFAS knowledge questionnaire (content knowledge): 20 true/false questions reduced to a total score. Total scores ranged from 0 to 20.
	End of intervention (11 months)		I: Communities of Practice n=17	12.1	14.1	p=0.14	
			C: Usual Practice n=17	10.4	10.8		
Di Noia 2003	Baseline	188 school personnel, community providers, and policy makers		Mean Scores:	Mean Scores:	F =25.67	Individual-item measures with Likert-scaled response options to determine if respondents knew where to locate drug abuse prevention findings and materials.
	Follow up (6 months)		I: Pamphlet n=55	0.94	1.04	p<0.05	
			I: CD-ROM n=64	0.96	0.75		Lower scores are indicative of more favourable ratings.
			I: Internet n=69	0.73	0.63		
Forsetlund 2003	Baseline	148 public health physicians	I: Workshop, information service, discussion list, free access to databases n=73	Mean Scores		Mean Difference	Baseline scores included in analysis. Scores were summed and means for individual overall scores computed.
	End of intervention (1.5 years)						
						SK: 0.4	
						t=4.3	
						95% CI (0.2-0.6)	Respondents graded self-perceived knowledge (SK) and knowledge about terms of importance to critical appraisal (CK) on scales ranging from 0 to 2 for CK and from 0 to 3 for SK. An additional question was added to concept knowledge, scored as either 0 or 1. Higher scores indicative of more favourable ratings.
				SK:1.1		p=0.00	
				CK:1.3		CK: 0.2	
						t=2.6	
						95% CI (0.0-0.3)	
						p=0.01	
			C: Access to free library services for one year n=75	SK:0.7			
				CK:1.1			

Note: I = Intervention C = Control.

Barwick et al. [21] was the third study that evaluated changes in knowledge by administering a 20-item true or false questionnaire measuring participants knowledge related to the use of an evidence based tool recently introduced into practice. Members in the community of practice group were defined as deliberate communities of people who share knowledge, learn together and create common practices supporting knowledge exchange among practitioners. Statistically significant between group differences were not reported between practitioners involved in an interactive communities of practice group versus usual practice.

#### Change in practice

***Randomized Controlled Trials*** All of the included studies evaluated change in practice (Table 2). Significant between group differences for changes in practice were only found in one of the five included studies by Dobbins et al. [5]. This study evaluated the effectiveness of KT strategies on evidence based decision making and the number of public health policies and programs implemented which documented the inclusion of research evidence. While a significant effect of any of the interventions was not shown on global EIDM (*p* < 0.45), in regards to effects on the number of public health policies and programs, health departments that received tailored and targeted messages plus access to an online registry of pre-processed research evidence improved significantly from baseline to follow-up (*p* < 0.01) [5]. This was in comparison to the groups that had access to the online registry only, or the groups that had access to the online registry and also had a knowledge broker working one on one with them. Also observed was that the use of knowledge brokers along with access to the online registry of pre-processed research evidence showed a trend towards a positive effect when organizational research culture was perceived at baseline as low. However, health departments with a low organizational research culture only benefited slightly when they received the tailored and targeted messaging plus access to the online registry, yet showed great improvements when the research culture was high [5]. Simply having access to an online registry of research evidence appeared to have no impact on evidence-informed decision making.

**Table 2:** Change in practice

**Randomized controlled trials (4)**							
**Study**	**Measurement period**	**Study population**	**Groups**	**Baseline**	**Follow up**	**Overall effect**	**Comments**
Barwick 2009	Baseline	34 Child & youth mental health practitioners	I: Communities of Practice n=17	Mean Scores	Mean Scores		
	End of intervention (12 months)			Use:	Use:	Use:	**Use**- 20-item questionnaire of self reported use of CAFAS implementation supports reduced to a total score. Responses were 'yes', 'no', or 'don't know/does not apply'.
				4.88	6.55	F=0.02	
						p=0.87	
				Change:	Change:	Change:	**Change**-10-question Likert scale of self- reported change reduced to a total practice change score. Items were rated as 'very much', 'somewhat', 'very little', or 'not at all'.
				3.00	8.81	F=0.20	
						p=0.65	
				Rating:	Rating:	Rating:	**Rating**: Total number of times clinicians rated the CAFAS in practice.
				NR	152	NR	
			C: Usual Practice n=17	Use:	Use:		
				4.88	4.22		
				Change:	Change:		
				1.33	1.80		
				Rating:	Rating:		
				NR	65		
Dobbins 2009	Baseline (2004)	108 public health departments in Canada	I: Tailored and targeted messaging	Mean Scores	Mean Scores		
				GIDM 5.61	GIDM 5.75	GIDM p < 0.45	**GIDM**-Global Evidence-Informed Decision Making- Mean self report score on the extent to which research evidence was considered in a recent program planning decision in the previous 12 months.
	End of Intervention (2006)		n=36	HPP 5.49	HPP 7.89	HPP < 0.01	
			I: Services of a knowledge broker	GIDM 5.45	GIDM 6.08		Responses ranged from: 1= not at all to 7= completely/
			n=36	HPP 6.53	HPP 6.03		
			C:Access to health evidence.ca registry	GIDM 5.43	GIDM 6.17		**HPP**-Public Health Policies and Programs Respondents asked whether the public health policies and programs were being implemented by their health department (yes/no). A ‘yes’ was coded as 01 and a ‘no’ was coded as a ‘02’. Total number was summed and compared across groups from baseline to post intervention.
				HPP 6.50	HPP 6.22		
			n=36				
Di Noia	Baseline	188 school personnel, community providers, and policy makers		Mean Score	Mean Score	NS	Frequency of searching for information
2003	Follow up (6 months)		I: Pamphlet n=55	1.56	1.60		Statistical test not reported. Lower scores are indicative of more favourable ratings
			I: CD-ROM n=64	1.53	1.48		
			I: Internet n=69	1.62	1.51		Internet was most effective intervention.
Forsetlund 2003	Baseline	148 public health physicians	I: Workshop, information service, discussion list, free access to databases n=73	Use of Research Percentage	Use of Research Percentage	NR	Statistical test not reported.
	End of intervention (1.5 years)			0%	0%		Analysis of the contents of local health service reports for use of research. Respondents sent in relevant documents analyzed by researchers. Scores for reports were recoded and reported as 'used' or 'not used' research.
			C: Free access to library services n=75	0%	1.3%		

Note: I = Intervention C = Control.

**Table 3:** Time series analysis (1)

**Study**	**Measurement period**	**Study population**	**Groups**	**Time (month/ year)**	**Intervention**	**Control**	**Overall effect**	**Measurement**
Hanbury	Baseline	93 community mental health professionals	I: Educational session (didactic presentation, peer discussion, group work on real life vignettes)	05/03	10	58	Intervention:	Monthly percentage adherence
2009	(2004)			06/03	23	75	NR	
				07/03	13	65	National	National event was modeled for the control and intervention site
				08/03	27	78	Event:	
			n = 49	09/03	42	72	(t = 3.28,	
			C: Usual Practice	10/03	35	62	P = 0.0001)	
				11/03	15	65		
			n = unstated	12/03	13	37		
				01/04	24	51		
				02/04	17	60		
				03/04	37	74		
				**04/04**	55	72		
				05/04	46	86		
				06/04	17	70		
				07/04	57	81		
				08/04	62	85		
				09/04	63	76		
				10/04	65	82		
				***11/04***	83	77		
				***12/04***	63	71		
				***01/05***	85	67		
				***02/05***	91	83		
				03/05	62	74		
				04/05	72	69		
				05/05	72	69		

Note: National event occurred 04/04 highlighted in bold. Intervention was delivered 11/04 to 02/05 highlighted in bold italics.

There were no statistically significant differences on the outcome change in practice on participants allocated to communities of practice in Barwick et al. [21] versus usual practice. While the KT strategies evaluated in Forsetlund et al. [23] and in Di Noia et al. [22] (described above) showed significant between group differences on the outcome knowledge, the KT strategies did not translate into significant changes in practice. Participants in Forsetlund et al. [23] who received the multi-faceted strategy related to EIDM showed no change in the use of research in written reports after the intervention.

#### Time Series Analysis

The time series analysis included in this review [24] evaluated the effectiveness of a theory of planned behaviour intervention implemented among community mental health professionals to improve adherence to a national suicide prevention guideline (Table 3). The KT strategy which consisted of an educational session comprised of didactic presentation, peer discussion, and group work on real life vignettes did not have a significant impact on adherence. During the course of the study two extraneous events occurred including: a national event where a guideline was introduced by the Health Care Commission which occurred in both intervention and control groups; and a local event causing a change in system for monitoring service-user-discharges which occurred in the intervention site only. Although the extraneous events made it difficult to isolate the effects of this from the intervention, using multiple time series analysis and including a control site, it was possible to model this into the analyses to estimate the impact of each of the events. Although the review authors did not focus on within group data, when comparing the intervention, local, and national event upon the change in the intervention group only, the national event had a statistically significant impact on adherence, *p* = 0.0001 [24].

## Discussion

Barriers and supports to research use can arise from various sources including: the practice environment, potential adopters and the evidence-based innovation [3,25,26]. Characteristics of these sources help provide insight into the impact of KT interventions tested.

### Characteristics of the interventions and providers

#### Exposure

Characteristics of the intervention, including exposure to the intervention, may have affected the extent to which the KT interventions resulted in knowledge or practice change among participants. For example, the educational session in Hanbury et al. [24] was only one day in length which may have been too short to have had an impact on participants' practice. Similarly, Dobbins et al. [5] report that there may have been discrepancies in the ability of the interventions to be implemented, with the rate of successful intervention differing across intervention groups. Given that 30% of participants allocated to the knowledge broker group had limited or no engagement it is possible that exposure to knowledge brokers may have been inadequate to affect practice.

Successful KT strategies may have increased exposure to the intervention, given that they were highly accessible or contained an element of tailoring responsive to the needs and preferences of providers. For example, the KT strategy in Di Noia et al. [22] did not require participants to physically travel anywhere or set aside a pre-specified time to review materials over the Internet, CD-ROM, or pamphlet thereby allowing participants to review materials at their own convenience. Materials were also sent out to participants by mail, fax, or email according to their preference and materials were tailored to include constituency specific content responsive to differing prevention needs. Post hoc analyses in the study by Di Noia et al. [22] favoured dissemination of materials via the Internet. This finding is supported in a recent meta-analysis of moderate quality examining Internet-based learning in the health professions. Internet-based learning was shown to be educationally beneficial and resulted in effects similar to those of traditional instructional methods [27]. A limitation of the meta-analysis however, was the pooling of disparate sources of evidence. Statistically significant differences were found favouring tutorials, longer-duration courses, and online peer discussion suggesting that an increased level of interaction may be beneficial. More studies are needed to investigate whether Internet based learning leads to actual and sustained change in practice.

Dobbins et al. [5] demonstrated in their study that KT interventions actively delivered and tailored to the needs of end users show promising results. The most effective KT strategy in their study was tailored, targeted messages which also employed content matching by ensuring the content of the message is relevant and applicable to the intended audience. This KT strategy was also actively delivered to decision makers rather than requiring them to access it independently. Providing a high level of accessibility plus tailoring the KT strategy to meet the personal needs of decision makers may lead to changes in knowledge and practice. The ability of tailored messaging to facilitate research use is supported by existing literature [28] as study participants show increased motivation to process information actively when they perceive the information to be personally relevant [29].

Forsetlund et al. [23] catered to the needs of participants to increase higher attendance at workshops. This was associated with greater effects in changing provider knowledge in a positive direction, however, the intervention did not translate to change in practice. One explanation may be that while workshops and educational sessions have been shown to modestly affect simple behaviours, they are less effective at changing complex behaviours [30].

#### Passive versus active interventions

Comparisons between passive versus interactive, and multi-component versus single interventions are often cited in the literature. Commonly reported findings, including a meta-analysis by Mansouri and colleagues [31,32], suggest that multi-component interventions have greater effects than single interventions. Differing results were found in this review. Simple or single KT strategies that included an active component assessed in this review were shown in some circumstances to be as effective as complex, multi-component ones when changing practice, a finding supported in a high quality systematic review by Grimshaw and colleagues [9,33]. This was evidenced in two of the five primary studies [5,22]. The highly interactive, multi-component interventions tested in both Forsetlund et al. [23] and Dobbins et al. [5] did not influence change in practice. The complexity of interventions may dilute the key messages of the intervention and reduce the ability of providers to understand or to acquire the information presented [5].

Passive strategies that were implemented unaccompanied with additional interventions were tested in two of the five studies and were ineffective. This finding is also frequently supported by existing literature [31,32,34-37]. Dobbins et al. [5] demonstrated that simply having access to a resource that repackaged review contents into a short summary of key findings, assessment of the methodological quality and recommendations, was not enough to influence evidence-informed decision making among public health practitioners. Systematic reviews have become widely recognized as a support to evidence-informed decision making in health care. The availability of a systematic review alone does not ensure that decision makers know it is available to them or can interpret the findings or use the evidence in service delivery decisions [38]. Further evaluations of these resources are needed to ensure users' needs and preferences are being met, to demonstrate their impact, justify their funding [39,40] and ensure the relevance and applicability of the results to the practice setting [41].

The second study evaluated the effectiveness of printed materials (pamphlet), another single and passive strategy. Pamphlets were less effective when compared with single but more active KT strategies including CD-ROM and Internet [22]. This finding is supported in a recent review by Farmer et al. [36] that reported when compared to no intervention, printed educational materials slightly improved professional behaviour but not patient outcomes. When dissemination of printed educational materials was compared to alternative interventions including educational initiatives; Farmer et al. concluded that they may slightly improve outcomes but there was not enough evidence to be certain. Grimshaw et al. [33] found slightly different results when they evaluated the effects of the dissemination of educational materials compared to audit and feedback and multi-component interventions involving educational outreach. Grimshaw et al. [33] concluded that because printed educational materials may lead to improvements in care, policy makers should not dismiss this strategy given its possible effect, low cost and feasibility in the health care system. The variation in study findings may be due to the characteristics of the KT strategy itself as important features of the information source including attractiveness, content, format, mode of delivery, timing, frequency, and complexity of targeted behaviour change are likely to have an effect on uptake [36].

### Characteristics of participants

Characteristics of participants in the studies comprising this review also varied which may have led to differences in the effectiveness of interventions. A high quality systematic review by Squires and colleagues [42] found that nurses' use of research is positively influenced by education (having a graduate degree compared to a bachelors degree or diploma); current role (leadership, advanced practice, clinical specialty compared to staff nurse); and job satisfaction. The results in this review did not consistently confirm this earlier research. For example, the KT strategy evaluated among well educated physicians with advance practice roles in Forsetlund et al. [23] did not positively influence research use. The sample in Barwick et al. [21] consisted of a wide range of individuals involved in child and youth mental health including social work, child and youth care, early education and one registered nurse. The level of education among this group varied from diploma or certification to graduate level education. The large differences in this group may have led to more variability in change in knowledge and practice scores among participants, due to differences in interest, willingness and ability to acquire new knowledge. The differences in effect found among the different studies, despite factors present that have been shown to positively influence research use, may be due to individual factors including attitudes toward research. Attitudes toward research was confirmed to positively affect research use in the same high quality review by Squires and colleagues [42] of individual characteristics related to research utilization. Lavis [39] reported on existing reviews examining the factors that influence the use of research evidence in policymaking and found that when there is harmony between research evidence and the beliefs, values, interests or political goals of policymakers, the use of research evidence is likely to increase. Several characteristics of the individual practitioner have been identified as being influential in the translation of research to practice [43]. Further research is required to investigate which individual characteristics of public health practitioners are associated with research utilization.

### Characteristics of the organizations

Finally, differences in the characteristics of the organizations may have also led to differences in the effectiveness of interventions. This is evidenced by the findings presented in Dobbins et al. [5] which revealed both positive and negative changes in the KT intervention's effectiveness when matched with organizational research culture.

It is obvious that contextual factors weigh heavily on the effectiveness of different interventions. Influences on professional behaviour are complex and are influenced by organizational and contextual variables that should be considered [44]. This suggests that several barriers may need to be assessed and overcome prior to implementing certain KT interventions. A shortage of knowledge exists regarding how organizational characteristics are related to the decision to implement evidence-based practices [45]. Wang et al. [45] surveyed county system leaders and found that their decision to implement an evidence-based program was influenced by their objective need for the program and by their perception of the county's organization climate and motivation to change. The importance of culture, a factor found to be influential on research use in this review, is also supported by existing research. Orton and colleagues [46] examined the use of research evidence by public health policy-makers in a systematic review and report one of the many barriers to use of research evidence included the culture in which policy-makers work. Additionally, in a systematic review by Meijers et al. [47] statistically significant relationships were found between research use and the role of the nurse, multi-faceted access to resources, organizational climate, multi-faceted support, time for research activities, and provision of education. These findings highlight the need for future research that examines organizational characteristics and how factors of systems or agencies including capacity, climate, culture and readiness to change affect research uptake.

### Strengths and limitations

There were several strengths and limitations of this review. Strengths include a comprehensive search strategy that was developed in accordance with expert opinion. We consider the included studies to be a relatively complete set of studies for the period 2000 to 2010. For this review, we used KT terms that were shown to have high and medium discriminatory power in the search strategy due to the difficulty in information retrieval related to the field of KT. Studies were obtained from multiple electronic databases, supplemented by hand searching and reference list checking of included articles and background papers for potentially relevant studies. This systematic review followed rigorous methodology including the use of two independent review authors to screen all studies for relevance and assessment of methodological quality of relevant studies. We also undertook detailed data extraction about the quality of the studies, characteristics of the studies, and interventions [19].

A limitation common in the KT literature was the quantity and quality of existing research related to this field of research. The review authors found a paucity of literature directed towards changing the knowledge, skills, or practice of public health practitioners despite increased societal pressure to increase capacity of EIDM among this group of health care providers. The quality of evidence included in this review was moderate (See Additional file 2).

The paucity of literature in this field of research combined with the high variation in described settings, interventions and outcome measures across studies made it difficult to synthesize and draw conclusions from this evidence base. In addition, a major limitation of this systematic review is the difficultly to disentangle whether the KT strategy itself was effective or whether it was in fact the context in which it was delivered. Recommendations related to interventions can therefore only be given or considered within the boundaries of the context they were delivered in.

A final limitation common in the KT literature and studies in public health is the use of self-report measures which are subject to recall bias, and often had unknown validity or reliability. In addition, there was inconsistency in the measures being used for the outcome of interest making it difficult if not impossible to build a consistent body of knowledge on which KT interventions influence changes in knowledge or practice. The development and testing of more objective data collection tools for measuring evidence-based practice is needed as limitations of current methods constrain the ability to validly measure research utilization [43].

## Conclusion

There is an imperfect evidence base to support decisions about which KT strategies are likely to be effective for increasing research use under different circumstances [33]. Due to differing characteristics of the users, the providers, the intervention and the organizations where the interventions may have been implemented, it is difficult to predict the effectiveness of KT interventions or suggest if their effectiveness will remain constant in differing contexts. Conclusions about interventions therefore cannot be taken on their own without considering the characteristics of the knowledge that was being transferred, the providers, participants and organizations.

Knowledge translation is a multidimensional concept that requires an understanding of its mechanisms, methods, and measurements, as well as its influencing factors at the individual and contextual levels, and the interaction between those levels [48]. While randomized controlled designs are the most rigorous designs for evaluating effectiveness, this design does not illuminate why certain KT interventions are successful or not. Other study designs including mixed methods and qualitative studies are valuable as they may increase understanding of the processes involved between program delivery and outcome [48]. In addition, a realist synthesis method, aimed at not only explaining how and why certain interventions work, but understanding the contexts in which they work best, may provide insight regarding "*complex social interventions which act on complex social systems*" [49]. We would also recommend that programmatic research in the area of research utilization in public health be undertaken to break this large research topic into smaller more manageable pieces allowing for more detailed analysis of different classes of determinants. This may help build a more coherent picture from smaller study findings.

The theory of planned behaviour based intervention tested by Hanbury and colleagues [24] was not effective at changing practice. Behaviour change interventions are commonly designed without evidence of a formal analysis of the target behaviour or the theoretically predicted mechanisms of action [50]. Behaviour interventions are often guided by implicit common sense models of behaviour that do not cover the full range of possible influences often excluding potentially important variables. Further research is needed to develop more comprehensive models and evaluate the effectiveness of existing ones that guide the development of behaviour change interventions.

It is essential that KT strategies in public health continue to be evaluated and their usefulness documented in the literature so that they can be adjusted or modified accordingly. The documented effectiveness of KT strategies in public health and the contexts in which they were delivered in can be utilized by practitioners whom wish to promote evidence-informed decision making among public health decision makers.

## Competing interests

The authors declare that they have no competing interests.

## Authors' contributions

DC and RL created the study concept, design and constructed and refined the search strategy. All titles and abstracts were screened independently. RL, JY and three undergraduate nursing students from McMaster University also contributed to the review of titles and abstracts. RL and JY independently conducted full text review of potentially relevant articles and quality assessment. Data extraction was done first by RL and reviewed by JY. Drafting of the manuscript and critical revision for important intellectual content was done by RL, JY, MD, DC and MB. RL wrote the final report and is the guarantor for the paper. All authors read and approved the final manuscript.

## Pre-publication history

The pre-publication history for this paper can be accessed here:

http://www.biomedcentral.com/1471-2458/12/751/prepub

## Supplementary Material

Additional file 1 ***Search Strategies.*** Table of data listing literature search strategies. Click here for file

Additional file 2 ***Risk of Bias Assessment of Included Studies.*** Results from the critical appraisal of included studies. Click here for file

Additional file 3 ***Characteristics of Included Studies.*** Table of data describing characteristics of included studies. Click here for file
