# Supplementary material for: The effectiveness of knowledge translation strategies used in public health: a systematic review
Source: BMC Public Health. 2012 Sep 7;12:751. doi: 10.1186/1471-2458-12-751 (PMC3532315; doi:10.1186/1471-2458-12-751)
Supplement: Additional file 1 — Search Strategies. Table of data listing literature search strategies. [file 1471-2458-12-751-S1.pdf]

Search strategy using highly discriminatory KT terms

**Electronic databases searched**	**Search terms used**	**Total hits**
CINAHL<br>(2000-Present)	1. clinical trials/<br>2. control$.tw.<br>3. random$.tw.<br>4. comparative studies/<br>5. experiment$.tw.<br>6. (time adj series). tw<br>7. impact.tw.<br>8. intervention$.tw.<br>9. evaluat$.tw.<br>10. effect?.tw.<br>11. exp pretest-posttest desgin/<br>12. exp quasi-experimental studies/<br>13. or/1-12<br>14. "cochrane database of systematic reviews".jn.<br>15. 13 not 14<br>16. author's strategy and 15<br><br>**AND**<br><br>Community OR Public Health*<br><br>**AND**<br>*Implementation*<br>*Adoption*<br>*Quality Improvement*<br>*Dissemination*<br>*Complex intervention:*<br>*Implementation (w/3) research*<br>*Complex intervention*<br>**Information**<br>**Change**<br>**Evaluation**<br>**Implementation**<br>**Utiliz/sation**<br>**Adoption**<br>**Quality improvement**<br>**Dissemination**<br>**Complex intervention:**<br>**Complex intervention**<br>**Instititionaliz/sation***	23430 (For high and medium discriminatory terms combined)<br><br>*Note:<br>*Italic terms* discriminate KT articles from non-KT articles for all KT papers.<br>**Bold terms** discriminate KT articles from non-KT articles for KT application papers only.

Search strategy using highly discriminatory KT terms

**Electronic databases searched**	**Search terms used**	**Total hits**
EMBASE<br>(2000-Present)	1. Randomized controlled trial/<br>2. random$.tw.<br>3. experiment$.tw.<br>4. (time adj series).tw.<br>5. (pre test or pretest or post test or posttest).tw.<br>6. impact.tw.<br>7. intervention$.tw.<br>8. chang$.tw.<br>9. evaluat$.tw.<br>10. effect?.tw.<br>11. compar$.tw.<br>12. control$.tw.<br>13. or/1-12<br>14. Nonhuman/<br>15 13 not 14<br>16. author's strategy and 15<br><br>**AND**<br><br>Community OR Public Health*<br><br>**AND**<br>*Implementation*<br>*Adoption*<br>*Quality Improvement*<br>*Dissemination*<br>*Complex intervention:*<br>*Implementation (w/3) research*<br>*Complex intervention*<br>**Information**<br>**Change**<br>**Evaluation**<br>**Implementation**<br>**Utiliz/sation**<br>**Adoption**<br>**Quality improvement**<br>**Dissemination**<br>**Complex intervention:**<br>**Complex intervention**<br>**Instititionaliz/sation***	36469<br>(For high and medium discriminatory terms combined)<br><br>*Note:<br>*Italic terms* discriminate KT articles from non-KT articles for all KT papers.<br>**Bold terms** discriminate KT articles from non-KT articles for KT application papers only.

Search strategy using highly discriminatory KT terms

**Electronic databases searched**	**Search terms used**	**Total hits**
MEDLINE<br>(2000-Present)	1. randomized controlled trial.pt.<br>2. random$.tw.<br>3. control$.tw.<br>4. intervention$.tw.<br>5. evaluat$.tw.<br>6. or/1-5<br>7. animal/<br>8. human/<br>9. 7 not (7 and 8)<br>10. 6 not 9<br>11. author's strategy and 10<br><br>**AND**<br><br>Community OR Public Health*<br><br>**AND**<br><br>*Implementation*<br>*Adoption*<br>*Quality Improvement*<br>*Dissemination*<br>*Complex intervention:*<br>*Implementation (w/3) research*<br>*Complex intervention*<br>**Information**<br>**Change**<br>**Evaluation**<br>**Implementation**<br>**Utiliz/sation**<br>**Adoption**<br>**Quality improvement**<br>**Dissemination**<br>**Complex intervention:**<br>**Complex intervention**<br>**Instititionaliz/sation***	30357<br>(For high and medium discriminatory terms combined)<br><br>*Note:<br>*Italic terms* discriminate KT articles from non-KT articles for all KT papers.<br>**Bold terms** discriminate KT articles from non-KT articles for KT application papers only.

Search strategy using highly discriminatory KT terms

Electronic databases searched	Search terms used	Total hits
The Cochrane Library Systematic Reviews (2000-Present)	Community OR Public Health*<br><br>**AND**<br><br>*Implementation*<br>*Adoption*<br>*Quality Improvement*<br>*Dissemination*<br>*Complex intervention:*<br>*Implementation (w/3) research*<br>*Complex intervention*<br>**Information**<br>**Change**<br>**Evaluation**<br>**Implementation**<br>**Utiliz/sation**<br>**Adoption**<br>**Quality improvement**<br>**Dissemination**<br>**Complex intervention:**<br>**Complex intervention**<br>**Instititionaliz/sation***	2292<br>(For high and medium discriminatory terms combined)<br><br><br>*Note:<br>*Italic terms* discriminate KT articles from non-KT articles for all KT papers.<br>**Bold terms** discriminate KT articles from non-KT articles for KT application papers only.

Search strategy using medium discriminatory KT terms

Electronic databases searched	Search terms used	Total hits
CINAHL<br>(2000-Present)	1. clinical trials/<br>2. control$.tw.<br>3. random$.tw.<br>4. comparative studies/<br>5. experiment$.tw.<br>6. (time adj series). tw<br>7. impact.tw.<br>8. intervention$.tw.<br>9. evaluat$.tw.<br>10. effect?.tw.<br>11. exp pretest-posttest desgin/<br>12. exp quasi-experimental studies/<br>13. or/1-12<br>14. "cochrane database of systematic reviews".jn.<br>15. 13 not 14<br>16. author's strategy and 15<br><br>**AND**<br><br>Community OR Public Health*<br><br>**AND**<br><br>*Change*<br>*Organiz/sational innovation*<br>*Innovation*<br>*Best practice:*<br>*Institutionali/sation*<br>*Diffusion of innovation*<br>*Translational research*<br>**Policy**<br>**Policies**<br>**Best practice:**<br>**Best practice**<br>**Continuing education**<br>**Implementation (w/3) research**<br>**Service innovation***<br>**Linkage and exchange***	23430 (For high and medium discriminatory terms combined)<br><br>*Note:<br>*Italic terms* discriminate KT articles from non-KT articles for all KT papers.<br>**Bold terms** discriminate KT articles from non-KT articles for KT application papers only.

Search strategy using medium discriminatory KT terms

Electronic databases searched	Search terms used	Total hits
EMBASE<br>(2000-Present)	1. Randomized controlled trial/<br>2. random$.tw.<br>3. experiment$.tw.<br>4. (time adj series).tw.<br>5. (pre test or pretest or post test or posttest).tw.<br>6. impact.tw.<br>7. intervention$.tw.<br>8. chang$.tw.<br>9. evaluat$.tw.<br>10. effect?.tw.<br>11. compar$.tw.<br>12. control$.tw.<br>13. or/1-12<br>14. Nonhuman/<br>15. 13 not 14<br>16. author's strategy and 15<br><br>**AND**<br><br>Community OR Public Health*<br><br>**AND**<br>*Change*<br>*Organiz/sational innovation*<br>*Innovation*<br>*Best practice:*<br>*Institutionali/sation*<br>*Diffusion of innovation*<br>*Translational research*<br>**Policy**<br>**Policies**<br>**Best practice:**<br>**Best practice**<br>**Continuing education**<br>**Implementation (w/3) research**<br>**Service innovation***<br>**Linkage and exchange***	36469<br>(High and medium discriminatory terms combined)<br><br>*Note:<br>*Italic terms* discriminate KT articles from non-KT articles for all KT papers.<br>**Bold terms** discriminate KT articles from non-KT articles for KT application papers only.

Search strategy using medium discriminatory KT terms

**Electronic databases searched**	**Search terms used**	**Total hits**
MEDLINE<br>(2000-Present)	1. randomized controlled trial.pt.<br>2. random$.tw.<br>3. control$.tw.<br>4. intervention$.tw.<br>5. evaluat$.tw.<br>6. or/1-5<br>7. animal/<br>8. human/<br>9. 7 not (7 and 8)<br>10. 6 not 9<br>11. author's strategy and 10<br><br>**AND**<br><br>Community OR Public Health*<br><br>**AND**<br>*Change*<br>*Organiz/sational innovation*<br>*Innovation*<br>*Best practice:*<br>*Institutionali/sation*<br>*Diffusion of innovation*<br>*Translational research*<br>**Policy**<br>**Policies**<br>**Best practice:**<br>**Best practice**<br>**Continuing education**<br>**Implementation (w/3) research**<br>**Service innovation***<br>**Linkage and exchange***	30357<br>(High and medium discriminatory terms combined)<br><br>*Note:<br>*Italic terms* discriminate KT articles from non-KT articles for all KT papers.<br>**Bold terms** discriminate KT articles from non-KT articles for KT application papers only.

Search strategy using medium discriminatory KT terms

**Electronic databases searched**	**Search terms used**	**Total hits**
The Cochrane Library Systematic Reviews (2000-Present)	Community OR Public Health*<br><br>**AND**<br><br>*Change*<br>*Organiz/sational innovation*<br>*Innovation*<br>*Best practice:*<br>*Institutionali/sation*<br>*Diffusion of innovation*<br>*Translational research*<br>**Policy**<br>**Policies**<br>**Best practice:**<br>**Best practice**<br>**Continuing education**<br>**Implementation (w/3) research**<br>**Service innovation***<br>**Linkage and exchange***	2292<br>(For high and medium discriminatory terms combined)<br><br><br>*Note:<br>*Italic terms* discriminate KT articles from non-KT articles for all KT papers.<br>**Bold terms** discriminate KT articles from non-KT articles for KT application papers only.
