# Supplementary material for: The effectiveness of knowledge translation strategies used in public health: a systematic review
Source: BMC Public Health. 2012 Sep 7;12:751. doi: 10.1186/1471-2458-12-751 (PMC3532315; doi:10.1186/1471-2458-12-751)
Supplement: Additional file 2 — Risk of Bias Assessment of Included Studies. Results from the critical appraisal of included studies. [file 1471-2458-12-751-S2.pdf]

Cochrane's Risk of Bias assessment of Randomized Controlled Trials (4)

**Study: Barwick 2009**		
**Entry**	**Judgment**	**Support for Judgment**
Random sequence generation<br><br>(selection bias)	Unclear<br><br>(Uncertain risk of bias)	Comment: The process by which the randomization occurred is not reported. Clinicians from 6 consenting organizations were randomly assigned, clustered by organization to either the intervention or control conditions (p. 20).
Allocation concealment (selection bias)	Unclear<br><br>(Uncertain risk of bias)	Comment: Although organizations were cluster randomized, there was insufficient information to permit judgment of 'Yes' or 'No.'
Blinding (participants)	No<br><br>(High risk of bias)	Comment: Blinding not done as per communication with author. Study was practice based related to real world practice change
Blinding (providers)	No<br><br>(High risk of bias)	Comment: Blinding not done as per communication with author.
Blinding (data collectors/outcome adjudicators)	No<br><br>(High risk of bias)	Comment: Blinding not done as per communication with author.
Blinding (data analysts)	No<br><br>(High risk of bias)	Comment: Blinding not done as per communication with author.
Incomplete outcome data addressed?	Unclear<br><br>(Uncertain risk of bias)	Comment: Insufficient information to permit judgment of 'Yes' or 'No'

Cochrane's Risk of Bias assessment of Randomized Controlled Trials (4)

**Study: Barwick 2009 (Continued)**		
**Entry**	**Judgment**	**Support for Judgment**
Free of selective reporting?	Yes<br>(Low risk of bias)	Comment: All outcomes identified a priori were reported on.
Free of other bias?	No<br>(High risk of bias)	• Risk of Co-Intervention: Other interventions to increase knowledge of EBP that the researchers were unaware of could have been occurring.<br>• Not all measurement tools were shown to be valid or reliable.<br>• Unclear if groups had similar baseline characteristics.<br>• Unclear if groups were similar in measurement of the outcome at baseline.

Cochrane's Risk of Bias assessment of Randomized Controlled Trials (4)

**Study: Di Noia 2003**		
**Entry**	**Judgment**	**Support for Judgment**
Random sequence generation (selection bias)	Unclear<br><br>(Uncertain risk of bias)	Comment: The process by which the randomization occurred is not reported.<br><br>Quote: "Randomly matched triads of sites. Random assignment."
Allocation concealment (selection bias)	Unclear<br><br>(Uncertain risk of bias)	Comment: Insufficient information to permit judgment of 'Yes' or 'No'.
Blinding (participants)	Unclear<br><br>(Uncertain risk of bias)	Comment: Insufficient information to permit judgment of 'Yes' or 'No'. Likely not done because of the nature of the intervention.
Blinding (providers)	Unclear<br><br>(Uncertain risk of bias)	Comment: Insufficient information to permit judgment of 'Yes' or 'No'. Likely not done because those developing the intervention materials would have knowledge of the intervention groups.
Blinding (data collectors/outcome adjudicators)	Yes<br><br>(Low risk of bias)	Comment: Insufficient information to permit judgment of 'Yes' or 'No'. Likely done because the outcome measurement was a survey completed by participants and therefore not likely to be influenced by lack of blinding.
Blinding (data analysts)	Unclear<br><br>(Uncertain risk of bias)	Comment: Insufficient information to permit judgment of 'Yes' or 'No'.

Cochrane's Risk of Bias assessment of Randomized Controlled Trials (4)

**Study: Di Noia 2003 (Continued)**		
**Entry**	**Judgment**	**Support for Judgment**
Incomplete outcome data addressed?	No<br>(High risk of bias)	Comment: Intention to treat analysis not completed.
Free of selective reporting?	Yes<br>(Low risk of bias)	Comment: All outcomes identified a priori were reported on.
Free of other bias?	No<br>(High risk of bias)	• Not all confounders considered at baseline measurement (years of experience/current position).<br>• Risk of Co-Intervention: Other interventions to increase knowledge of EBP that the researchers were unaware of could have been occurring.<br>• Data collection tools were not demonstrated to be valid or reliable.

Cochrane's Risk of Bias assessment of Randomized Controlled Trials (4)

**Study: Dobbins 2009**		
**Entry**	**Judgment**	**Support for Judgment**
Random sequence generation (selection bias)	Yes<br>(Low risk of bias)	Comment: Although this sequence is not truly random, risk of introducing bias using these methods is low.<br>Quote: " health departments were randomly allocated to groups in equal numbers within strata by computer-generated pseudorandom draws using standard algorithms" (p. 3).
Allocation concealment (selection bias)	Yes<br>(Low risk of bias)	Comment: Unlikely to foresee allocation assignment through the use of computer generated draws.
Blinding (participants)	Unclear<br>(Uncertain risk of bias)	Comment: Insufficient information to permit judgment of 'Yes' or 'No'. Likely not done because of the nature of the intervention.
Blinding (providers)	Unclear<br>(Uncertain risk of bias)	Comment: Insufficient information to permit judgment of 'Yes' or 'No'. Likely not done because those delivering the interventions would have knowledge of the intervention groups.
Blinding (data collectors/outcome adjudicators)	Yes<br>(Low risk of bias)	Comment: Data collectors were not aware of the groups to which participants had been allocated.
Blinding (data analysts)	Yes<br>(Low risk of bias)	Comment: Statistician did not have access to participant information and was not aware in the results set of who had been allocated to which groups.

Cochrane's Risk of Bias assessment of Randomized Controlled Trials (4)

**Study: Dobbins 2009 (Continued)**		
**Entry**	**Judgment**	**Support for Judgment**
Incomplete outcome data addressed?	Yes<br><br>(Low risk of bias)	Comment: Analysis was based on the initial treatment intent.<br><br>Quote: "Allows for flexible handling of missing data."(p. 7).
Free of selective reporting?	Yes<br><br>(Low risk of bias)	Comment: All outcomes identified a priori were reported on.
Free of other bias?	No<br><br>(High risk of bias)	• Risk of Co-Intervention: Other interventions to increase knowledge of EBP that the researchers were unaware of could have been occurring.<br><br>• Not all measurement tools were shown to be valid or reliable.

Cochrane's Risk of Bias assessment of Randomized Controlled Trials (4)

**Study: Forsetlund 2003**		
**Entry**	**Judgment**	**Support for Judgment**
Random sequence generation (selection bias)	Yes<br><br>(Low risk of bias)	Quote: “Enrolled physicians were subsequently randomized to one of two groups by an independent researcher using computer software” (p.5).
Allocation concealment (selection bias)	Yes<br><br>(Low risk of bias)	Comment: Computer software was used.
Blinding (participants)	Unclear<br><br>(Uncertain risk of bias)	Comment: Insufficient information to permit judgment of ‘Yes’ or ‘No’. Likely not done because of the nature of the intervention ,
Blinding (providers)	Unclear<br><br>(Uncertain risk of bias)	Quote: Insufficient information to permit judgment of ‘Yes’ or ‘No’. Likely not done because of the nature of the intervention
Blinding (data collectors/outcome adjudicators)	Yes<br><br>(Low risk of bias)	Quote: “Registrar of questionnaire data was blinded to group allocation.” “Researchers who scored the other study outcomes were blinded to the allocation of participants and whether the results were pre or post tests” (p.5).
Blinding (data analysts)	Unclear<br><br>(Uncertain risk of bias)	Comment: Insufficient information to permit judgment of ‘Yes’ or ‘No’.

Cochrane's Risk of Bias assessment of Randomized Controlled Trials (4)

**Study: Forsetlund 2003 (Continued)**		
**Entry**	**Judgment**	**Support for Judgment**
Incomplete outcome data addressed?	Yes<br><br>(Low risk of bias)	Quote: “Data for all responding participants were analyzed on an intention to treat basis, in the sense that even responders who had not received the intervention in full were included in the analysis” (p.5).
Free of selective reporting?	Yes<br><br>(Low risk of bias)	Comment: All outcomes identified a priori were reported on.
Free of other bias?	No<br><br>(High risk of bias)	• Baseline characteristics revealed a possible imbalance for some variables (sex, number of years as a public health physician, specialist status, previous exposure to courses in critical appraisal and number of reports written).<br>• Unclear if groups were similar in measurement of the outcome at baseline.<br>• Participants were asked to sign a contract about what they would change in their practice prior to follow up.<br>• Risk of Co-Intervention: In the time period evidence based practice was discussed in other public health settings which could have influenced the general level of knowledge.<br>• Not all measurement tools were shown to be valid or reliable.

EPOC Risk of Bias for Interrupted Time Series Design (1)

**Study: Hanbury 2009**		
**Entry**	**Judgment**	**Support for Judgment**
Was the intervention independent of other changes?<br><br>(Protection against secular changes)	Yes<br><br>(Low risk of bias)	Comment: Used control site; recorded and accounted for other events in the analysis including the introduction of guideline by Health Care Commission and at the intervention site only a change in system for monitoring service-user-discharges.
Was the shape of the intervention effect pre-specified?	Yes<br><br>(Low risk of bias)	Comment: The point of analysis is the point of intervention. Two extraneous events were also analysed and the point of their occurrence clearly identified.
Was the intervention unlikely to affect data collection?	No<br><br>(High risk of bias)	Comment: Used different data collection methods at Phase 1 and Phase 3. Phase 1 used interviews and Phase 3 used chart audits.
Was knowledge of the allocated interventions adequately prevented during the study?	Yes<br><br>(Low risk of bias)	Comment: Outcome measures were objective.
Were incomplete outcome data adequately addressed?	Unclear<br><br>(Uncertain risk of bias)	Comment: Insufficient information to permit judgment of ‘Yes’ or ‘No’.

Risk of Bias Tables for Interrupted Time Series Design (1)

**Study: Hanbury 2009 (Continued)**		
**Entry**	**Judgment**	**Support for Judgment**
Was the study free from selective outcome reporting?	Yes<br><br>(Low risk of bias)	Comment: All outcomes identified a priori were reported on.
Was the study free from other risks of bias?	No<br><br>(High risk of bias)	• No random allocation<br>• Only 1 control and 1 intervention site<br>• Researcher developed tool was used to measure outcomes.<br>• Baseline characteristics of the intervention and control group were not reported<br>• Unclear if outcomes assessed blindly
