# Supplementary material for: The effectiveness of knowledge translation strategies used in public health: a systematic review
Source: BMC Public Health. 2012 Sep 7;12:751. doi: 10.1186/1471-2458-12-751 (PMC3532315; doi:10.1186/1471-2458-12-751)
Supplement: Additional file 3 — Characteristics of Included Studies. Table of data describing characteristics of included studies. [file 1471-2458-12-751-S3.pdf]

Randomized Controlled Trials (4)

**Study**	**Authors**: Barwick, M.A., Peters, J. Boydell, K.<br><br>**Date**: 2009<br><br>**Country**: Canada
**Objective**	To examine whether practitioners in a community of practice (CoP) changed their practice more readily and demonstrated greater knowledge of the Child and Adolescent Functional Assessment Scale (CAFAS) than practitioners given access to the implementation supports typically available
**Methods**	**Design**: Randomized controlled trial<br><br>**Recruitment**: Fourteen Children's Mental Health service provider organizations newly added to the provincial CAFAS user group were invited to participate in the study. Participants were reimbursed for their travel and funds were provided to the participating organizations to secure clinical back-up to cover clinicians' absences.<br><br>**Inclusion/exclusion**: Children's mental health practitioners working in service provider organizations who agreed to participate in the study. All clinicians were eligible to participate in the study after they were trained in 2-day reliability and 1-day software orientation training and achieved interrater reliability on the CAFAS tool.<br><br>**Allocation**: Clinicians from 6 consenting organizations were randomly assigned, clustered by organization, to either the CoP or practice as usual PaU support conditions.
**Participants**	**Total Sample**: N= 34 participants completed baseline measurements<br><br>**Intervention group**: Communities of practice (CoP) n= 17<br><br>**Control group**: Practice as usual practice (PaU) n= 17

Randomized Controlled Trials (4)

**Study: Barwick et al. (2009) Continued**	
**Participants (continued)**	**Characteristics**: Participants were child and youth mental health practitioners working in publicly funded community based service provider organizations in Ontario. Participants were mostly female (89.2%), and had on average 9 years of experiences as a clinician (7 years among PaU group; 10.8 years among CoP group). Four participants had graduate level education, 8 had bachelors level training, 14 had diplomas or certifications in social work, social service work, child and youth care, or early childhood education, and there was one registered nurse (7 participants did not provide level of education data).<br><br>**Loss to follow-up**: 14 lost to follow up (6 in study group; 8 in control)<br><br>**Study duration**: 1 year 2006-2007
**Intervention**	**Interventions:** Community of practice-Established group of people sharing knowledge, learning together, and creating common practices.<br><br>**Description of Intervention**:<br><br>Session 1: The facilitator explained the purpose of the CoP is to support and develop the practice surrounding the use of the CAFAS tool. Participants were oriented to the various roles that help set-up, develop, nurture, and sustain the community, and set the stage for its sustainability. Members worked together and participated actively. There was also a key role for a content expert, who acted as a resource to the community when needed.<br><br>Sessions 2-6: Group invited to shape the agenda for the meetings. Conversation built in which advice, opinions, and information were offered, again situated in practice. Productive inquiry initiated the actions of knowledge access, knowledge exchange, and knowledge creation. The knowledge needed and shared was triggered by a real situation connected to practice.

Randomized Controlled Trials (4)

**Study: Barwick et al. (2009) Continued**	
**Intervention**	**Description of Control group**: Practitioners in the PaU group were given access to the implementation supports typically available.<br><br>**Intervention Duration:** 11 months<br><br>**Intervention Frequency:** CoP practitioners met as a 'community' of new CAFAS users 6 times over an 11 month period.<br><br>**Provider(s)**:Meetings were hosted and facilitated by the CAFAS Trainer<br><br>**Site:** Meetings were held in the same location<br><br>**Follow up**: End of intervention (11 months)<br><br>**Theoretical Framework:** Structure was developed according to certain key principles of Community of Practice models
**Outcomes**	Change in Knowledge<br>Change in Practice

**Study: Barwick et al. (2009) Continued**	
**Outcome Measurement Tool**	**Knowledge**: CAFAS knowledge questionnaire- (Content Knowledge) - 20 true/false questions measuring specific knowledge related to clinical use of the CAFAS scale reduced to a total CAFAS knowledge score. Total scores ranged from 0 to 20. Validity and reliability not reported.<br><br>**Practice**: 20-item questionnaire regarding respondents self reported use of CAFAS implementation supports reduced to a total CAFAS supports score. Responses were 'yes', 'no', or 'don't know/does not apply' Validity and reliability not reported.<br><br>**Practice**: 10-question Likert scale questionnaire to assess the degree of self-reported change reduced to a total practice change score. Items were rated as 'very much', 'somewhat', 'very little' or 'not at all'. Validity and reliability not reported.<br><br>**Practice**: Total number of times clinicians rated the CAFAS in practice. Validity and reliability not reported
**Study limitations**<br><br>(Items mentioned by review authors not already identified in risk of bias assessment)	**Study Authors:**<br><br>• Small sample size followed over a short duration<br><br>• Frequency of CAFAS ratings does not take into account variation in the number of patients entering into treatment in each organization<br><br>• CoP clinicians were provided with financial support<br><br>**Review Authors**:<br><br>• Convenience sample of organizations<br><br>• Baseline measurements were taken after session 1<br><br>• Low exposure to CoP sessions-average participation 3.7 out of 6 sessions

Randomized Controlled Trials (4)

**Study**	**Authors**: Di Noia, J., Schwinn, T.M., Dastur, Z.A., Schinke, S.P.<br><br>**Date**: 2003<br><br>**Country**: United States
**Objective**	To evaluate the effectiveness of three dissemination strategies (Pamphlets, CD-ROM, Internet) related to prevention program materials.
**Methods**	**Design**: Randomized controlled trial<br><br>**Recruitment:** Three adolescent substance abuse prevention programs were identified and illustrative dissemination materials were compiled for each. These materials were disseminated to school personnel, community providers, and policy makers. First by mailed letter invitation, then by telephone follow-up, sites were offered the opportunity to participate in the study.<br><br>**Inclusion/Exclusion:** Sites included schools, community agencies, policy making bodies and youth services agencies. Sites agreeing to participate were asked to identify professionals on staff to complete assessments at planned intervals and to review materials for three youth-oriented substance abuse prevention programs.<br><br>**Allocation:** Grouped by site, consenting professionals were stratified and matched on their constituency (school, agency, policy-making body) and geographic location. Matched triads of sites were randomly assigned to one of three arms: pamphlet, CD-ROM, or Internet.

Randomized Controlled Trials (4)

**Study: Di Noia et al. (2003) Continued**	
**Participants**	**Total Sample**: N=188 professionals<br><br>**Intervention groups**:<br><br>Pamphlet n=55<br><br>CD-ROM n=64<br><br>Internet n=69<br><br>**Characteristics**: The participants were professionals employed in schools, community agencies, and policy-making bodies. Schools were defined as public and independent educational facilities at the middle and junior high levels.<br><br>Community agencies were defined as private non-profit organizations that provide youth with human services including school dropout, delinquency, and pregnancy prevention; day treatment, juvenile probation and parole; educational tutoring; and recreational, neighbourhood, and club activities.<br><br>Policy-making organizations were government legislative, analytic, funding, and regulatory bodies that were at least in part dedicated to the provision or recommendation of drug abuse prevention services for youth.<br><br>Professionals included teachers, social workers, and other management and executive-level personnel who exercised decision-making power over the selection and application of adolescent drug abuse prevention programs. Respondents from target constituencies tended to be female, between the ages of 30 and 49 years, white, and well educated with close to half of respondents (48%) holding graduate degrees.<br><br>**Loss to follow-up**: Unstated<br><br>**Study Duration:** 2 years

Study: Di Noia et al. (2003) Continued	
**Intervention**	**Interventions:** Printed materials and information in CD-ROM or internet format tailored to prevention needs<br><br>**Description of Intervention**: Information was synthesized about three youth-oriented substance abuse prevention programs and a common presentation format for delivering this content via pamphlet, CD-ROM, and Internet was developed.<br><br>Materials described the rationale, strategies, and costs to prevent drug abuse, and the roles of schools, professionals, and community groups, and relevant private and government bodies in addressing this problem. Materials were tailored to be responsive to their differing prevention needs. Constituency-specific content was delivered to respondents in the CD-ROM and Internet arms.<br><br>Following receipt of completed pre-tests, professionals in the respective study arms were sent the pamphlet, CD-ROM, or logon name, password, and instructions for Internet access.<br><br>**Description of Control:** No control group<br><br>**Intervention Duration**: Participants had 6 months to review materials before first follow up measurement took place<br><br>**Intervention Frequency**: Independent study of materials<br><br>**Provider(s):** Researchers disseminated materials<br><br>**Site**: Unstated<br><br>**Follow up:** 6 and 12 months after receiving dissemination materials, participants completed post-test and follow-up measurements.<br><br>**Theoretical Framework:** Unstated
**Outcomes**	Change in Knowledge<br>Change in Practice

Randomized Controlled Trials (4)

**Study: Di Noia et al. (2003) Continued**	
**Outcome Measurement Tool**	**Knowledge:** Individual-item measures with Likert-scaled response options to determine where to locate drug abuse prevention findings and material. Lower scores indicative of more favourable ratings. Validity and reliability not reported.<br><br>**Practice**: Frequency with which respondents searched for prevention program materials was measured. Lower scores indicative of more favourable ratings. Validity and reliability no reported.
**Study Limitations**<br><br>(Items mentioned by review authors not already identified in risk of bias assessment)	**Study authors:**<br><br>• Limited generalizability due to small sample<br>• Unable to permit subgroup analyses of interactions among channel, constituency and program<br>• Interventions were slightly outdated<br>• The use of self reported single-item outcome measures<br>• Brief follow up periods<br><br>**Review authors:**<br><br>• Convenience sample<br>• Difficult to assess exposure to interventions due to the nature of independent study of materials<br>• Group of participants were well educated (half masters prepared) limiting generalizability of findings<br>• Could not use measure "Likelihood of requesting/implementing programs as a concrete measure of behaviour change

Randomized Controlled Trials (4)

**Study**	**Authors**: Dobbins, M., Hanna, S.E., Ciliska, D., Manske, S., Cameron, R., Mercer, S.L., O'Mara, L., DeKorby, K., Robeson, P.<br><br>**Date**: 2009<br><br>**Country**: Canada
**Objective**	To evaluate the effectiveness of three knowledge translation and exchange strategies in the incorporation of research evidence into public health policies and programs.
**Methods**	**Design**: Randomized controlled trial<br><br>**Recruitment:** After consent obtained from senior person in public health departments, name of person most directly responsible for making decisions about healthy body weight promotion identified and contacted via letter and follow up phone call<br><br>**Inclusion/exclusion:** All public health departments in Canada were eligible to participate identified through provincial databases.<br><br>**Allocation**: Participating health departments were stratified according to size of population served and randomly allocated to one of three intervention groups in equal numbers within strata by computer generated pseudorandom draws using standard algorithms
**Participants**	**Total Sample**: N= 108 public health departments<br><br>**Intervention groups**:<br><br>Targeted and Tailored Messaging (TM) n=36 Targeted and tailored messaging plus access to registry<br><br>Knowledge Broker (KB) n=36 Services of a knowledge broker plus access to registry and targeted and tailored messaging<br><br>**Control group:**<br><br>Health Evidence (HE) n=36 Access to healthevidence.ca registry

Study: Dobbins et al. (2000) Continued	
**Participants (continued)**	**Characteristics**: Participants were from participating regional and local public health departments in Canada and were directly responsible for making program decisions related to healthy body weight promotion in children. This included program managers and/or coordinators in Ontario, and program directors in the rest of Canada. Participation by province and territory ranged from 29% to 100% with the sample consisting primarily of health departments serving both urban and rural populations (46%).<br><br>**Loss to follow-up**:<br><br>Intervention: (TM) n=6 (KB) n=7<br><br>Control: (HE) n=7<br><br>Follow-up data were collected from 88 of 108 (81.5%) participating public health departments<br><br>**Study duration:** 2 years<br><br>Baseline assessment was completed September-November 2004, with the intervention taking place during the calendar year of 2005 when all interventions were introduced simultaneously. Post intervention assessment was completed January-March 2006.

Randomized Controlled Trials (4)

**Study: Dobbins et al. (2000) Continued**	
**Intervention**	**Description of Intervention:**<br><br>**TM group**: Tailored, targeted messages plus access to health-evidence.ca<br><br>Over seven successive weeks, on the same day each week and the same time of day, participants in the TM group were sent an email indicating that a systematic review related to healthy body weight promotion in children was available in full text at the link provided.<br><br>Participants received access to the PDF version of the systematic review, the published abstract of the review, as well as the short summary written. The text of the message was worded to say, 'this message is number XX in a series of seven emails you will receive on healthy body weight promotion in children as part of the KTE strategy you are being exposed to in this randomized controlled trial.<br><br>**KB group**: Included both the HE and TM components and a KB who worked one on one with decision makers in the public health departments. The KBs were Master's prepared, had extensive knowledge and expertise in public health decision making, as well as an understanding of the research process.<br><br>Specific tasks conducted by the KB included: ensuring relevant research evidence related to healthy body weight promotion was transferred to the public health decision makers in ways that were most useful to them, assisting them to develop the skill and capacity for evidence-informed decision making, and assisting them in translating evidence into local practice. Approximately twenty percent of KB time was spent facilitating knowledge and skill development either through face-to-face interaction such as workshops or online strategies such as webinars, interactive web enabled meetings, or conferences. Eighty percent of the brokers' time was spent preparing for and directly interacting with participants.

Randomized Controlled Trials (4)

**Study: Dobbins et al. (2000) Continued**	
**Intervention (continued)**	**Description of Control**:<br>HE group: Least interactive KTE strategy. HE group had access to health-evidence.ca which is a repository of systematic reviews evaluating any public health intervention. All participants in the study received electronic communication about the availability of this site. Upon searching this site for reviews evaluating strategies to promote healthy body weight in children, those in the HE group would have become aware of the title, citation, and assessment of the methodological quality of seven systematic reviews evaluating the effectiveness of interventions to promote healthy body weight in children. Participants in the HE group also had access to the published abstracts, and the full text articles and a short summary for each of the systematic reviews, written by the research team, with key findings and recommendations for public health policy and practice directly applicable to the types of decisions for which the participants were responsible.<br><br>**Duration of Intervention:** 1 year<br><br>**Frequency of Intervention:** Varied<br><br>**Providers:** Researchers, Professionals<br><br>**Site:** Workplace<br><br>**Follow up**: End of intervention<br><br>**Theoretical Framework**: Framework for Research Dissemination and Utilization
**Outcomes**	Change in Practice

Randomized Controlled Trials (4)

**Study: Dobbins et al. (2009) Continued**	
**Outcome Measurement Tool**	Telephone-administered survey (knowledge transfer and exchange data collection tool). Reported reliability 0.65 Cronbach alpha.<br><br>**Practice**: Global Evidence-Informed Decision Making- Mean self report score on the extent to which research evidence was considered in a recent program planning decision in the previous 12 months. Responses ranging from one (not at all) to seven (completely).<br><br>**Practice**: Public Health Policies and Programs- Respondents asked whether the public health policies and programs were being implemented by their health department (yes/no). The total number was summed.
**Study Limitations**<br><br>(Items mentioned by review authors not already identified in risk of bias assessment)	**Study authors:**<br><br>• Self-reported outcome measures<br><br>• Participants may have not been aware of all public health policies and programs provided by their organization leading to both under and over reporting of this outcome<br><br>• Variable exposure to intervention- Up to 30% of participants did not engage with the KB at all or to a limited extent<br><br>• Participants who completed baseline measurements were different in follow up surveys in 30% of departments<br><br>**Review authors:**<br><br>• Questionnaire only reported as satisfactory Cronbach alpha of 0.65<br><br>• Not described how exposure to knowledge broker was estimated<br><br>• Using two different knowledge brokers could have led to differences between groups using that intervention

Randomized Controlled Trials (4)

**Study**	**Authors**: Forsetlund, L. Bradley, P., Forsen, L., Nordheim, L., Jamtvedt, G., Bjørndal, A.<br><br>**Date**: 2003<br><br>**Country**: Norway
**Objective**	The aim of this study was to evaluate whether a tailored theory-based and multifaceted intervention targeted at the whole process of evidence-based practice increased the explicit integration of research in public health physicians' decision-making
**Methods**	**Design**: Randomized controlled trial<br><br>**Recruitment:** The invitation letters explained that project participants would have free access to a library service. In return, they would be asked to return questionnaires and examples of written reports to be used for programme evaluation. Participants were also informed that some would be asked to co-operate further during the project period. Recruitment was stopped when 73 had been allocated to the intervention group and 75 to the control group, fulfilling the number of the sample size calculations.<br><br>**Inclusion/exclusion**: All public health physicians working in municipalities in Norway with more than 3000 inhabitants (N = 332) were invited to participate in the project.<br><br>**Allocation:** Public health physicians were enrolled by the primary author upon receipt of the consenting letter. Enrolled physicians were subsequently randomized to one of two groups by an independent researcher using computer software.

Study: Forsetlund et al. (2003) Continued	
**Participants**	**Total Sample**: N=148<br><br>**Intervention group**: n=73<br><br>**Control group** n=75<br><br>**Characteristics**: Participants were public health physicians working in municipalities in Norway. Public health physicians in Norway are geographically scattered; one physician in each of the country's 435 municipalities. The sample was physicians who were predominately male, were on average 47 years of age and had been working in the field on average 12 years in the intervention group vs 9.5 years in the control group, working experience in rural and urban settings. More physicians in the intervention group had previously attended sessions in critical appraisal.<br><br>**Loss to follow-up**:<br>Analysed in intervention group: Questionnaire 58 (79%) Reports 17 (23%)<br>Analysed in control group: Questionnaire 61 (81%) Reports 25 (33%)<br><br>**Study Duration**: January 1999 to January 2001.
**Intervention**	**Interventions:** workshop, information service, discussion list, access to databases<br><br>**Description of Intervention:** The intervention program was intended to lead the participants from the first knowledge stage to the confirmation stage when adoption was to occur based on innovation-diffusion process.<br><br>**Workshop**: Interactive small-group setting involving small group problem-based activities and discussion. Involved posing and formulating questions, searching skills, critical appraisal and practical application of research evidence in practice.<br><br>**Goal-Setting Contract**: Physicians were asked to state three things that they would change when returning to practice.

Study: Forsetlund et al. (2003) Continued	
**Intervention**	**Information Services (including library access)**: Included on-going support, access to several databases and consisted of: a question and answer service where upon submitting a questions physicians would receive references or reports based on relevant studies found; access to course material and how to practice evidence-based public health; and links to other sources of information on evidence-based practice.<br><br>**Discussion List**: Discussion stimulated by giving general reminders, providing and asking for feedback and allocating peer discussion. Providers announced when reports had been written and critically appraised selected articles. Participants were reminded of ongoing support services.<br><br>**Newsletters**: Three newsletters reported on principles of evidence-based health care and project activities, including feedback on database use.<br><br>**Description of Control**: Participants in the control group received free access to library services for one year.<br><br>**Intervention Duration**: April 1999 until the end of January 2001<br><br>**Intervention Frequency:** 11 courses on evidence-based public health varying from 1-5 days to maximize attendance<br>3 newsletters<br><br>**Provider**: Two public health physicians and two librarians<br><br>**Site**: Web-based and workshop format<br><br>**Follow up**: Follow-up measurements were started immediately at the end of the intervention<br><br>**Theoretical Framework**: Rogers' model of innovation diffusion

Randomized Controlled Trials (4)

**Study: Forsetlund et al. (2003) Continued**	
**Outcomes**	Change in Knowledge<br>Change in Practice
**Measurement (Screening) Tool**	Baseline scores included in analysis.<br><br>**Knowledge:** Questionnaire measured self-perceived concept knowledge (scale 0 to 2) and self perceived source knowledge (scale 0 to 3). An additional question was added to concept knowledge, scored as either 0 or 1. Concept knowledge was knowledge of importance to critical appraisal and source knowledge was information about sources for evidence based practice.<br><br>Content Knowledge: Mean of additive score of 0 = 'unknown', 1 ='known', 2 = 'so known that I can explain to others' + an extra point (1) if correctly answering "Method chapter" as to what is the most important chapter for deciding scientific quality of an article.<br>Source Knowledge: Mean of additive score of 0 = 'unknown', 1 = 'known, but not used', 2 = 'read', 3 = 'used in a public health decision-making situation'.<br><br>Scores were summed and means for individual overall scores computed. Higher scores indicative of more favourable ratings. The analysis of internal consistency of scale items based on the 55 pilot test data yielded a Cronbach alpha score ranging from 0.83 to 0.87.<br><br>**Practice:** Analysis of the contents of local health service reports for use of research. Respondents sent in relevant documents analyzed by two assessors. Scores for reports were recoded and reported as 'used' or 'not used' research. The weighted Kappa scores for interrater agreement on use of research information for reports were 0.50, 0.91 and 0.87 at pretest respectively and 0.89, 0.75 and 0.74 at post-test.

Randomized Controlled Trials (4)

**Study: Forsetlund et al. (2003) Continued**	
**Study Limitations**<br><br>(Items mentioned by review authors not already identified in risk of bias assessment)	**Study authors:**<br><br>• Low statistical power<br>• Unreliability of measures and treatment implementation<br>• Low response rate for post-tests<br>• Increased effort to obtain more documents could have been made during data collection<br>• Possible that intervention was not adequately implemented in terms of teaching methods and duration<br>• 1.5 years may have been too short a time perspective<br>• Risk of co-intervention-In the time period evidence based practice was discussed in other public health settings influencing the general level of knowledge<br>• Experiment group could guess the hypothesis to a greater extent than control<br>• Sample contained innovators or early adopters<br><br>**Review authors:**<br><br>• Per communication with author measure of change in practice only collected at post-test (telephone survey/postal survey/self reported searching of Cochrane and Medline)<br>• Per communication with author, hypothetical assignment was not included as a measure of practice, decision to adopt included items measuring intention

Time Series Analysis (1)

**Study**	**Authors**: Hanbury, A., Wallace, L., Clark, M.<br><br>**Date**: 2009<br><br>**Country**: England
**Objective**	To test the effectiveness of a Theory of Planned Behaviour intervention implemented among community mental health professionals to improve adherence to a national suicide prevention guideline.
**Methods**	**Design**: Interrupted Time series design<br><br>**Recruitment:** All community mental health professionals in the intervention site were invited to participant. The intervention site was an NHS Trust in the West Midlands. Audit data was collected from an anonymous alternative control site where no intervention occurred.<br><br>**Inclusion/exclusion:** Unstated<br><br>**Allocation**: N/A
**Participants**	**Total Sample**: N=93 community mental health professionals<br><br>**Intervention group**: n =49 attended educational session<br><br>**Control group**: n= unclear<br><br>**Characteristics**: Community mental health professionals in the West Midlands region of the UK. Included community psychiatric nurses, psychiatrist, and occupational therapists.<br><br>**Loss to follow-up**: 28 lost to follow up (21 returned questionnaire post educational session)<br><br>**Study duration**: 2002-2006

Time Series Analysis (1)

<table>
<tr><th colspan="2">Study: Hanbury et al. (2009) Continued</th></tr>
<tr><td>Intervention</td><td>Intervention: Educational session (comprised of didactic presentation, peer discussion, group work on real life vignettes)

Description of Intervention: Educational session comprised three components designed to target normative beliefs.

First component: a presentation that contained factual statements, statistics and graphs taken from key Department of Health publications highlighting and supporting the guideline evidence base. The presentation was designed to convey positive normative beliefs that all staff adhere to the guideline and expect other staff to adhere.

Second component: group discussion facilitated to ensure that positive normative beliefs were emphasized and any negative normative beliefs challenged.

Third component: comprised group work on two real life vignettes developed in consultation with the professional head of nursing: one depicting an episode of care in which the guideline had been adhered to and a near-miss for a service-user avoided, and one in which the guideline had not been adhered to and there had been a negative outcome.

Providers: Training coordinators'

Site: Conducted at each community mental health teams' base

Length of Intervention: One day

Follow up: Intervention delivered from November, 2004 to February 2005 (Phase 2); adherence data collected until May 2006 (Phase 3)

Description of Control: Practice as usual

Theoretical Framework: Theory of Planned Behaviour</td></tr>
</table>

Time Series Analysis (1)

<table>
<tr><th colspan="2">Study: Hanbury et al. (2009) Continued</th></tr>
<tr><td>Outcomes</td><td>Change in Practice</td></tr>
<tr><td>Outcome Measurement Tool</td><td>Practice: Monthly percentage adherence recorded in the intervention and control site</td></tr>
<tr><td>Study Limitations

(Items mentioned by review authors not already identified in risk of bias assessment)</td><td>Study Authors:
Some discontinuity occurred between those who returned the questionnaire and those who attended the intervention
Staff turnover was a problem at the intervention site
Through using the audit adherence data aggregated across the mental health directorate it was not being possible to break the data down to the level of the individual health professionals
The timing of the local event made it difficult to isolate the effects of this from the intervention
Review Authors:
How sites were picked is not addressed
Unclear who control group participants were
Procedure for outcome measurement not stated
Could not use data related to questionnaire because measured "intention"</td></tr>
</table>
